# P-937. Impact of Rapid Carbapenemase Testing on Time to Active Therapy

**DOI:** 10.1093/ofid/ofaf695.1140

**Published:** 2026-01-11

**Authors:** Kellie Fortier, Jessica Chamberlin, Jared Cvetko, Lindsey A Johnson, Leonard Vande Kerkhoff, Rachael Craft

**Affiliations:** Banner Health, Phoenix, AZ; Banner Health, Phoenix, AZ; Banner University Medical Center - Tucson, Tucson, Arizona; Banner Health, Phoenix, AZ; Banner Health, Phoenix, AZ; Banner Health, Phoenix, AZ

## Abstract

**Background:**

Antimicrobial resistance continues to be a worldwide threat. Data from the Center for Disease Control (CDC) and our health system shows carbapenem-resistant Enterobacterales (CRE) are a growing problem in the US and in Arizona; within our health system the incidence of CREs have been increasing since 2020. At the end of 2023, rapid carbapenemase testing with NG-TEST® CARBA5 was implemented. This study evaluated if implementation of the NG-TEST® CARBA-5 assay improved time to active therapy

**Figure:**
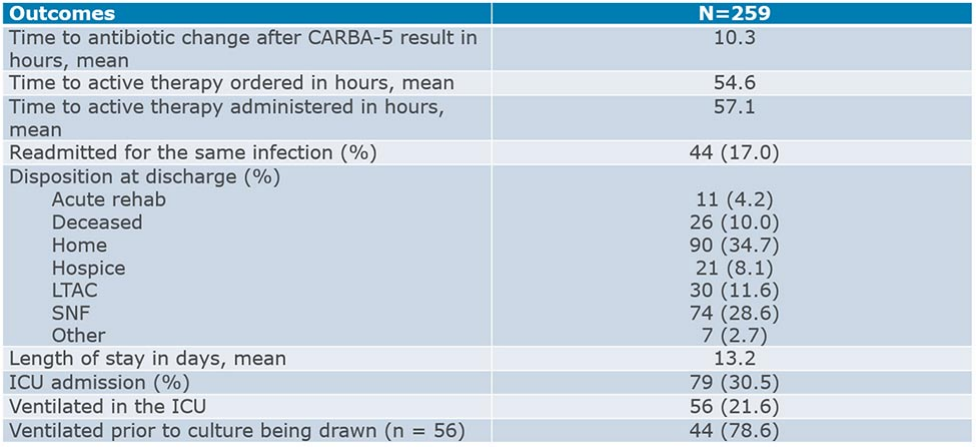
Primary and Secondary Endpoints

**Methods:**

This descriptive, retrospective analysis included admitted patients with an Enterobacterales positive culture, which exhibited non-susceptibility to a carbapenem and underwent CARBA5 testing between 3/1/24-6/30/24. The primary outcome was time to active therapy prescribed. Other outcomes included time to active therapy administered, in-hospital mortality, readmission for the same infection within 90 days, average length of stay (LOS), and intensive care unit (ICU) admission or transfer.

**Results:**

A total of 259 patients were included. New Delhi metallo-β-lactamase producing *K. pneumoniae* from a urinary source was the most common CRE. Time from culture drawn to active treatment ordered was 54.6 hours. In hospital mortality was 10% percent with a readmission rate of 17%. Mean LOS was 13.2 days with 30.5% of patients admitted or transferred to an ICU. Approximately 24% of patients never received active therapy, with 77% of those cultures being from urine. A previous system evaluation showed time to active antimicrobial therapy prescribed was 97.8 hours with an in-hospital mortality of 16%.

**Conclusion:**

Implementation of CARBA5 testing resulted in a reduction in time to active therapy prescribed by almost 2 days compared to a previous evaluation. In hospital mortality was lower after initiation of CARBA5 testing but could not be statistically compared as groups differed on inclusion and exclusion criteria. A 2-day reduction in time to active therapy is clinically relevant. Further work within the health system is being done to improve time to active therapy. This evaluation serves as a useful source of data for future evaluations or interventions.

**Disclosures:**

All Authors: No reported disclosures

